# Steroidal and Phenolic Glycosides from the Bulbs of *Lilium pumilum* DC and Their Potential Na^+^/K^+^ ATPase Inhibitory Activity

**DOI:** 10.3390/molecules170910494

**Published:** 2012-09-03

**Authors:** Zhong-Liu Zhou, Zong-Cai Feng, Chun-Yan Fu, Hua-Lin Zhang, Jing-Min Xia

**Affiliations:** 1 Chemistry Science and Technology School, Zhanjiang Normal University, 29 Cunjin Road, Zhanjiang 524048, China; Email: zhanghualing303@163.com (H.L.Z.); xiajingming88@163.com (J.-M.X.); 2 Development Center for New Materials Engineering Technology in Universities of Guangdong, Zhanjiang Normal University, 29 Cunjin Road, Zhanjiang 524048, China; Email: fengzongcai921@163.com; 3 Department of Pharmacy, Shaoyang Medical College Level Speciaity School, 18 Baoqing Road, Shaoyang 422000, China; Email: chunyanfull@yahoo.com.cn

**Keywords:** *Lilium pumilum* DC, pumilum A, Na^+^/K^+^ ATPase

## Abstract

A new steroidal saponin, named pumilum A (**1**), and a new phenolic glycoside, *threo*-1-(4′-hydroxy-2′-methoxyphenyl)-2-(2′′,4′′-dihydroxyphenyl)-1,3-propanediol-4′-*O*-*β*-D-glucopyranoside (**7**) were isolated from the methanolic extract of the bulbs of *Lilium pumilum* DC, along with five known steroidal saponins. Their chemical structures were elucidated on the basis of detailed spectroscopic analysis, including 1D and 2D NMR techniques and chemical methods. In addition, the inhibitory activity of all the isolates on Na^+^/K^+^ ATPase was evaluated.

## 1. Introduction

“Bǎi-hé”, a Chinese crude drug, is prepared from the bulbs of the *Lilium* species and is regularly used nowadays in China as a sedative, antitussive and anti-inflammatory agent [1,2,3,4]. Previously, phenolic glycosides, steroidal saponins and steroidal alkaloids have been isolated from the bulbs of the *Lilium* species [5,6,7,8,9,10,11,12]. However, to date, a survey of the literature showed that no chemical work has been done on *Lilium pumilum* DC.

**Figure 1:**
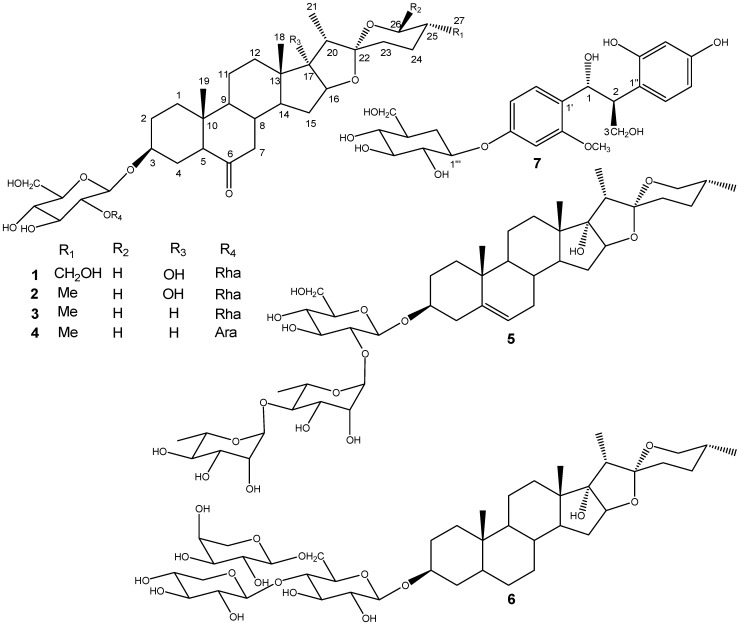
Structures of compounds **1**–**7** isolated from the bulbs of *Lilium pumilum* DC.

Our studies indicated that the methanolic extract of the bulbs of *Lilium pumilum* DC showed significant Na^+^/K^+^ ATPase inhibition activity (IC_50_ value: 0.5 × 10^−5 M^). To further investigate the constituents and screen the bioactive constituents from its bulbs, a phytochemical study was performed that resulted in the isolation of new compounds **1** and **7**, along with five known steroidal saponins. In the present article, we describe the structural elucidation of new compounds **1** and **7**, together with the Na^+^/K^+^ ATPase inhibitory activity of all these compounds **1**–**7** (Figure 1).

## 2. Results and Discussion

The methanolic extract from the bulbs of *Lilium pumilum* DC was successively subjected to column chromatography over silica gel, ODS, Diaion HP-20 and semipreparative HPLC to afford six steroidal saponins and a new phenolic glycoside. All compounds were obtained from this plant for the first time.

Compound **1** had the molecular formula C_39_H_62_O_15_, as deduced from the positive-ion HR-ESI-MS (*m*/*z* 793.6704 [M+Na]^+^) and ^13^C-NMR spectrum. The glycosidic nature of **1** was indicated by the strong absorption bands at 3435 cm^−1^ and 1045 cm^−1 in^ the IR spectrum. The typical absorptions at 975, 915, 895, and 860 cm^−1^ in the IR spectrum suggested that **1** was a spirostanol saponin [13,14]. The intensity of the absorptions (895 > 915) indicated that the absolute configuration of C-25 was *R*. The ^1^H-NMR spectrum contained signals for two oxygenated methylene proton signals at δ 4.58 (2H, d, *J* = 7.8 Hz, H-26), 3.74 (1H, dd, *J* = 10.6, 5.5 Hz, H-27a) and 3.67 (1H, dd, *J* = 10.7, 7.2 Hz, H-27b), two anomeric proton signals at δ 6.13 (1H, br s, H-1″) and 5.11 (1H, d, *J* = 7.2 Hz, H-1′), as well as four methyl proton signals at δ 0.82 (3H, s), 0.91 (3H, s), 1.21 (3H, d, *J* = 7.0 Hz), and 1.81 (3H, d, *J* = 6.8 Hz). The signal at δ 1.81 was due to the methyl group of rhamnose (Table 1). The ^13^C-NMR and DEPT spectrum showed a quaternary carbon signal at δ 109.7, which is the characteristic C-22 of a spirostanol skeleton [15,16]. The presence of a carbonyl group in **1** was established from the IR (1707 cm^−1^) and ^13^C-NMR spectra (δ 209.4). The existence of D-glucose and L-rhamnose were determined by hydrolysis of **1** with 1 M HCl and GC analysis. The *J* values of the anomeric proton signals indicated the *β*- and α-configuration at the anomeric centers of D-glucose and L-rhamnose, respectively. These findings showed that **1** was a (25*R*)-spirostanol diglycoside.

**Table 1:** ^13^C and ^1^H-NMR data of **1** in pyridine-*d_5_* (400 MHz for H, 100 MHz for C).

NO.	C	H	NO.	C	H
1	36.8	1.56 (1H, m)	20	44.7	2.21 (1H, q, *J* = 7.2 Hz)
		1.08 (1H, m)	21	9.7	1.21 (3H, d, *J* = 6.8 Hz)
2	29.3	1.84 (1H, m)	22	109.7	
		1.37 (1H, m)	23	31.5	1.92 (1H, m)
3	76.0	3.99 (1H, m)			1.45 (1H, m)
4	26.4	2.21 (1H, m)	24	24.1	2.17 (1H, m)
		1.75 (1H, m)			1.38 (1H, m)
5	56.4	2.26 (1H, m)	25	39.2	2.03 (1H, m)
6	209.4		26	64.0	4.58 (2H, d, *J* = 7.8 Hz)
7	46.9	2.54 (1H, m)	27	64.5	3.74 (1H, dd, *J* = 11, 5.5 Hz)
		2.42 (1H, m)			3.67 (1H, dd, *J* = 11, 7.2 Hz)
8	38.1	1.83 (1H, m)	Glc		
9	53.7	1.37 (1H, m)	1′	99.2	5.11 (1H, d, *J* = 7.2 Hz)
10	40.8		2′	79.5	4.22 (1H, m)
11	21.2	1.34 (1H, m)	3′	78.4	4.30 (1H, m)
		1.57 (1H, m)	4′	72.1	4.15 (1H, m)
12	32.0	2.13 (1H, m)	5′	78.2	3.86 (1H, m)
		1.49 (1H, m)	6′	62.8	4.33 (1H, m)
13	45.8				4.49 (1H, m)
14	53.2	2.17 (1H, m)	Rha		
15	32.0	2.17 (1H, m)	1″	102.4	6.13 (1H, br s)
		1.46 (1H, m)	2″	72.6	4.68 (1H, m)
16	89.8	4.42 (1H, m)	3″	72.9	4.37 (1H, m)
17	89.9		4″	74.2	4.39 (1H, m)
18	17.3	0.82 (3H, s)	5″	69.5	4.81 (1H, m)
19	13.2	0.91 (3H, s)	6″	18.7	1.81 (3H, d, *J* = 6.8 Hz)

**Figure 2:**
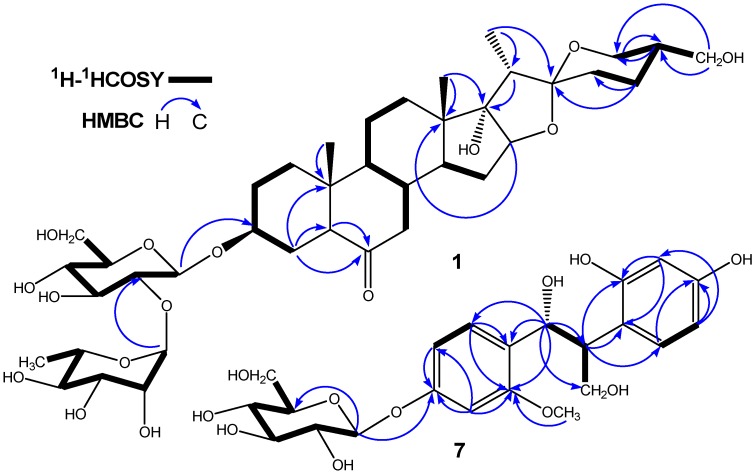
Key HMBC and ^1^H-^1^H COSY correlations of **1** and **7**.

**Figure 3:**
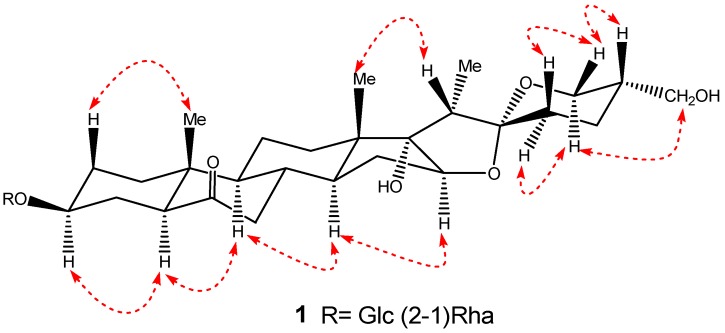
Selected ROESY correlations of **1**.

Comparison of the ^1^H and ^13^C-NMR spectra of **1** with those of **2** indicated that **1** contained a same sugar chain as **2**, the difference between these two saponins only appearing in the F-ring of their aglycons. The ^1^H and ^13^C-NMR spectra of **1** clearly displayed that C-27, which was presented as a methyl in **2** [^1^H-NMR: δ 0.71 (3H, d, *J* = 5.4 Hz); ^13^C-NMR: *δ* 17.3 (Me)], was modified to a hydroxymethyl in **1** [^1^H-NMR: δ 3.74 (1H, dd, *J* = 11, 5.5 Hz) and 3.67 (1H, dd, *J* = 11, 7.2 Hz); ^13^C-NMR: δ 64.5 (-CH_2_OH)]. The chemical shift values of C/H-27 and detailed 2D-NMR analysis of COSY, ROESY and HMBC correlations also implied that the aglycone moiety of **1** had one more hydroxyl group attached at C-27 (Figure 2 and Figure 3). On the basis of the above evidence, the structure of **1** was formulated as (25*R*)-3*β*,17*α*,27-triol-spirostan-6-one 3-*O*-*α*-L-rhamnopyranosyl-(1→2)-*β*-D-gluco- pyranoside, and it was named pumilum A.

Compound **7** had the molecular formula C_22_H_28_O_11_, deduced from the negative-ion HR-ESI-MS (*m*/*z* 467.1936 [M−H]^−^) and ^13^C-NMR spectrum, which exhibited 16 carbon signals due to the aglycone, in addition to six carbon signals of a β-D-glucopyranosyl group. The ^1^H and ^13^C-NMR spectra of **7** showed the presence of two aromatic ABX systems [δ (H) 7.55 (1H, d, *J* = 8.4 Hz, H-6′), 7.16 (1H, d, *J* = 8.0 Hz, H-6′′), 7.04 (1H, dd, *J* = 2.0, 8.4 Hz, H-5′), 7.08 (1H, d, *J* = 2.0 Hz, H-3′ ), 6.69 (1H, d, *J* = 2.0 Hz, H-3′′), and 6.51 (1H, dd, *J* = 2.0, 8.0 Hz, H-5′′); δ (C) 161.8 (C-4′′), 161.5 (C-2′′), 159.9 (C-4′), 157.3 (C-2′), 132.8 (C-6′), 125.6 (C-6′′), 119.9 (C-1′′), 114.8 (C-1′), 111.2 (C-5′), 106.7 (C-5′′), 105.8 (C-3′), and 97.4 (C-3′′)] (Table 2), suggesting the presence of two 1, 2, 4-trisubstituted phenyls in **7**. The detailed 2D-NMR analysis of ^1^H-^1^H COSY, HMQC, and HMBC correlations also suggested that the aglycone moiety of **7** had two 1, 2, 4-trisubstituted phenyls (Figure 2). In the aliphatic region, the ^1^H-NMR spectra of **7** were completely identical to those of 1, 2-bis(4-hydroxy-3-methoxyphenyl)-1,3-propandiol 4′-*O*-β-D-glucopyranoside [17], ABMX-type signals were clearly observed [including an oxygen-bearing methylene at δ 4.27 (2H, dd, *J* = 9.8, 5.0 Hz, H-3), an oxygen-bearing methine at δ 5.56 (1H, d, *J* = 7.4 Hz, H-1), and a methine at δ 3.50 (1H, td, *J* = 7.4, 5.0 Hz, H-2)]. In combination with the presence of three aliphatic carbon signals [δ 66.8 (C-3), 78.9 (C-1), and 44.9 (C-2)] in the ^13^C-NMR spectrum of **7** supported the presence of the aliphatic 1,3-propanediol chain. Additionally, the ^1^H and ^13^C-NMR spectra of **7** displayed the presence of one glucopyranosyl unit [δ (H) 5.71 (1H, d, *J* = 7.6 Hz, H-1′′′), δ (C) 102.4 (C-1′′′)], as well as a methoxyl group [δ (H) 3.66 (3H, s), δ (C) 55.6]. The HMBC spectrum of **7** exhibited long-range correlations from H-1 (*δ* 5.56) to C-2 (*δ* 44.9), C-3 (*δ* 66.8), C-1′ (*δ* 114.8), C-2′ (*δ* 157.3), C-6′ (*δ* 132.8) and C-1′′ (*δ* 119.9); and from H-2 (*δ* 3.50) to C-1 (*δ* 78.9), C-3 (*δ* 66.8), C-1′ (*δ* 114.8), C-1′′ (*δ* 119.9), C-2′′ (*δ* 161.5), and C-6′′ (*δ* 125.6), respectively, indicated the locations of the two phenyls linked to the aliphatic chain, 1,3-propanediol. In addition, the HMBC spectra of **7** revealed the methoxyl group (δ 3.67) was coupled to aromatic carbon C-4′′ (δ 161.8), suggesting that the methoxyl groups was linked to C-4′′ in **7** (Figure 2). Thus, the aglycone of **7** was believed to be 1-(4′-hydroxy-2′-methoxyphenyl)-2-(2′′,4′′-dihydroxyphenyl)-1, 3-propanediol.

**Table 2:** ^13^C and ^1^H-NMR data of **7** in pyridine-*d_5_* (500 MHz for H, 125 MHz for C).

NO.	C	H	NO.	C	H
1	78.9	5.56 (1H, d, *J* = 7.4 Hz)	3′′	97.4	6.69 (1H, d, *J* = 2.0 Hz)
2	44.9	3.50 (1H, td, *J* = 7.4, 5.0 Hz)	4′′	161.8	
3	66.8	4.27 (2H, dd, *J* = 9.8, 5.0 Hz)	5′′	106.7	6.51 (1H, dd, *J* = 2.0, 8.0 Hz)
1′	114.8		6′′	125.6	7.16 (1H, d, *J* = 8.0 Hz)
2′	157.3		1′′′	102.4	5.71 (1H, d, *J* = 7.6 Hz)
3′	105.3	7.08 (1H, d, *J* = 2.0 Hz)	2′′′	74.9	4.27 ^a^
4′	159.9		3′′′	79.0	4.11 (1H, m)
5′	111.2	7.04 (1H, dd, *J* = 2.0, 8.4 Hz)	4′′′	71.2	4.26 ^a^
6′	132.8	7.55 (1H, d, *J* = 8.4 Hz)	5′′′	78.4	4.43 (1H, m)
1′′	119.9		6′′′	62.5	4.31 (1H, m)
2′′	161.5		2′-CH_3_O	55.6	3.67 (3H, s)

^a^ Overlapped signals.

The *J* value (7.6 Hz) of the anomeric proton implied the β-configuration of the glucose moiety. The *β*-D-glucopyranosyl group was attached to the C-4′ position of the aglycone, which was supported by a long-range correlation between the anomeric proton signal (*δ* 5.71) of the *β*-D-glucopyranosyl group and the C-4′ signal (*δ* 159.9) in the HMBC spectrum of **7**. The relative stereochemistry of C-1 and C-2 of **7** was determined to be *threo* by comparing the coupling constant H-1 and H-2 (*J* = 7.4 Hz) with those of the *erythro* and *threo* isomers [18,19]. Therefore, **7** was assigned as *threo*-1-(4′-hydroxy-2′-methoxyphenyl)-2-(2′′, 4′′-dihydroxyphenyl)-1, 3-propanediol-4′-*O*-*β*-D-glucopyranoside.

The structures of the other isolated components, namely (25*R*)-3β,17α-diol-5α-spirostan-6-one 3-*O*-α-L-rhamnopyranosyl-(1→2)-β-D-glucopyranoside (**2**), (25*R*)-3β-hydroxyl-5α-spirostan-6-one 3-*O*-α-L-rhamnopyranosyl-(1→2)-β-D-glucopyranoside (**3**), (25*R*)-3β-hydroxy-5α-spirostan-6-one 3-*O*-α-L arabinopyranosyl-(1→6)-β-D-glucopyranoside (**4**), dioscin (**5**), and (25*R*)-5α-spirostan-3β,17α-diol 3-*O*-β-D-xylopyranosyl-(1→4)-[α-L-arabinopyranosyl-(1→6)]-β-D-glucopyranoside (**6**) were determined by comparison to the ^1^H and ^13^C-NMR spectral data in the literature [2,20,21,22,23].

The inhibitory activity of **1**–**7** on Na^+^/K^+^ ATPase was assayed. Ouabain was used as a positive control (IC_50_ value: 0.1 × 10^−5 M^). Saponins **3** and **4** were found to inhibit sensitive Na^+^/K^+^ ATPase with the IC_50_ values of 7.3 × 10^−5^ and 9.1 × 10^−6^ M, respectively, while the others were inactive [IC_50_ > 1.0 × 10^−4^ M]. Thus, this research suggested that the saponins from the methanolic extract may be primary Na^+^/K^+^ ATPase inhibitors. The weak Na^+^/K^+^ ATPase inhibitory activity of the initial extract (compounds **1**–**7**) is thus presumably due to other as yet unidentified compounds. One possible reason for this is that compounds with highly Na^+^/K^+^ ATPase inhibitory activity are low in the BuOH-soluble fraction of the methanolic extract from the bulbs of *Lilium pumilum* DC. Another possible reason is compounds with increased activity may be found in other extraction fractions (ethyl acetate extract, chloroform extract and petroleum ether extract) of the methanolic extract from the bulbs of this medicine plant. Therefore, more chemical work needs to be done in the future.

## 3. Experimental

### 3.1. General

Specific rotation measurements were recorded on a Perkin-Elmer 242 MC polarimeter. UV spectra were recorded on a Hewlett-Packard HP-845 UV-VIS spectrophotometer. IR spectra were recorded on a Nicolet 470 spectrometer and MS on a Varian MAT-212 mass spectrometer and a Shimadzu GC-MS model QP2010 Plus spectrophotometer, respectively. NMR spectra were recorded on a Bruker AM-400 spectrameter (400 MHz for ^1^H-NMR) or a Bruker DRX-500 (500 MHz for ^1^H-NMR) spectrameter using standard Bruker pulse programs. Chemical shifts are given as δ values with reference to tetramethylsilane (TMS) as internal standard. Column chromatography separations were carried out on silica gel (200–300 mesh, Qingdao Haiyang Chemical Co. Ltd, Qingdao, China), ODS (50 mesh, AA12S50, YMC), Diaion HP-20 (Pharmacia, Peapack, NJ, USA) and Sephadex LH-20 (Pharmacia, Peapack, NJ, USA). Ouabain sensitive dog kidney Na^+^/K^+^ ATPase was obtained from Sigma (Shanghai, China). All other chemicals used were of biochemical reagent grade.

### 3.2. Plant Material

The bulbs of *Lilium pumilum* DC were collected in Lanzhou, Gansu Province of China in September 2010, and were identified by one of the authors (Chun-Yan Fu of Department of Pharmacy, Shaoyang Medical College Level Speciaity School, Shaoyang). A voucher specimen (NO. 20100908) has been deposited in the authors’ laboratory.

### 3.3. Extraction and Isolation

Fresh bulbs of *Lilium pumilum* DC (5 kg) were cut into pieces and extracted with methanol (3 × 8 L, 3h each) under reflux. The solvent was removed at reduced pressure to give a viscous residue (328 g). The entire crude extract was suspended in H_2_O (3 L), and extracted with *n*-BuOH five times (3 L each) to give an *n*-BuOH extract (206 g). This extract was then fractionated by silica gel column chromatography (column: 90 cm × 9 cm) using a mobile phase composed of CHCl_3_-MeOH (9:1–6:1–4:1–2:1–1:1, *v*/*v*), and finally with MeOH alone to afford six fractions [Fr.1 (98 mg), Fr.2 (11.2 g), Fr.3 (37.8 g), Fr.4 (11.1 g), Fr.5 (14.3 g), and Fr.6 (51.6 g)]. Fr.2 (11.2 g) was chromatographed on silica gel (column: 70cm × 4 cm) eluting with CHCl_3_-MeOH-H_2_O (40:10:1) and ODS silica gel with MeOH-H_2_O (4:1) and MeCN-H_2_O (1:1) to furnish **3** (14.5 mg), **4** (16.8 mg), **5** (16.3 mg), and **6** (20.7 mg). Fr.1 (98 mg) was passed through a Diaion HP-20 column with a gradient mixture of MeOH-H_2_O and the MeOH eluate fraction was subjected to silica gel column eluting with CHCl_3_-MeOH-H_2_O (40:10:1; 30:10:1) and ODS silica gel with CH_3_CN-H_2_O (1:1–5:7–1:2) to give **2** (15.6 mg) and **7** (17.2 mg) as the pure compounds, and **1** with a few impurities. Final purification of **1** (19.4 mg) was accomplished by semipreparative HPLC. The method was an isocratic gradient of 70% methanol in H_2_O over 15 min, and then followed by 95% methanol for 5 min (flow rate: 3 mL/min).

### 3.4. Characterization of Pumilum A (***1***)

Obtained as a white amorphous powder, 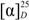: −88.7° (*c* 0.048, MeOH); UV λ_max_ (MeOH): 279 nm; IR *v*_max_ (KBr): 3435, 2945, 2880, 1707, 1455, 1375, 1245, 1205, 1155, 1080, 1045, 985, 975, 915, 895, 860, 805, 710 cm^−1^. HR-ESI-MS *m*/*z* 793.6907 (C_39_H_62_O_15_Na [M+Na]^+^, Cal. 793.6904). ^1^H-NMR and ^13^C-NMR (pyridine *d_5_*) data see Table 1.

### 3.5. Characterization of Threo-1-(4′-hydroxy-2′-methoxyphenyl)-2-(2′′,4′′-dihydroxy-phenyl)-1,3-propanediol-4′-O-β-D-glucopyranoside (***7***)

Obtained as an amorphous powder. 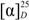: −76.3° (*c* 0.5, MeOH). UV (MeOH) λ_max_: 282 nm. IR *v*_max_ (KBr): 3433, 3391, 1630, 1601, 1495, 1470, 1441, 1386, 1345, 1253, 1202, 1172, 1143, 1041, 955, 860, 826 cm^−1^. HR-ESI-MS *m*/*z* 467.1936 ([M−H]^−^, Calcd. for C_22_H_27_O_11_, 467.1903). ^1^H-NMR and ^13^C-NMR (pyridine *d_5_*) data see Table 2.

### 3.6. Acid Hydrolysis of Compound ***1***

A solution (2.5 mg) of **1** in 1 M HCl (1 mL) was heated at 100 °C for 2 h under an N_2_ atmosphere. After cooling, the solution was removed by blowing with N_2_. The residue was dissolved in a solution of 1-(trimethylsilyl) imidazole (0.5 mg) in pyridine (1.0 mL), and stirred at 60 °C for 5 min. After removal of the solvent with a stream of N_2_, the residue was partitioned between H_2_O and CH_2_Cl_2_ (1:1 *v*/*v*). The CH_2_Cl_2_ fraction was analyzed by GC using a L-Chirasil-Val column (0.32 mm × 25 m). The temperature of the injector and detector were 200 °C. A temperature gradient system was used for the oven, starting at 100 °C for 1 min and increasing up to 180 °C at a rate of 5 °C/min. The peaks of the hydrolysates of **1** were confirmed by comparison of retention times of authentic samples D-glucose, L-rhamnose treated with 1-(trimethylsilyl) imidazole [24].

### 3.7. Assay of Na^+^/K^+^ ATPase Activity

The Na^+^/K^+^ ATPase activity was assayed according to the reported method with some modification [25]. The reaction mixture composed of 50 mM Tris-HCl (pH 7.3, 37 °C), 3 mM ATP, 4 mM Mg^2+^, 130 mM Na^+^, 20 mM K^+^, and 0.02 units of Na^+^/K^+^ ATPase, with or without test compound dissolved in dimethyl sulfoxide (DMSO), was incubated for 20 min at 37 °C. The concentration of DMSO in the reaction mixture was held at 5%. The reaction was terminated by addition of 50% CCl_3_COOH. The released inorganic phosphate was determined by the following method. To the test solution was added 0.5% sodium dodecyl sulfate, 0.1% 2,4-diaminophenol 2 M HCl in 1% Na_2_SO_3_ and 1% ammonium heptamolybdate in 1 M H_2_SO_4_. After 25 min, the absorbance at 660 nm was recorded.

## 4. Conclusions

Two new compounds, pumilum A (**1**) and *threo*-1-(4′-hydroxy-2′-methoxyphenyl)-2-(2′′,4′′-dihydroxyphenyl)-1,3-propanediol-4′-*O*-*β*-D-glucopyranoside (**7**) were isolated from the the bulbs of *Lilium pumilum* DC, along with five known steroidal saponins. Compounds **3** and **4** exhibited weak inhibitory activity on Na^+^/K^+^ ATPase with the IC_50_ values of 7.3 × 10^−5^ and 9.1 × 10^−6 M^, respectively.

## Supplementary Materials

Supplementary materials can be accessed at: http://www.mdpi.com/1420-3049/17/9/10494/s1.
