# Supplementary figures and images for: Steroidal and Phenolic Glycosides from the Bulbs of Lilium pumilum DC and Their Potential Na+/K+ ATPase Inhibitory Activity
Source: Molecules. 2012 Sep 3;17(9):10494–502. doi: 10.3390/molecules170910494 (PMC6268628; doi:10.3390/molecules170910494)

**Figure S1. Compounds 1–7.**

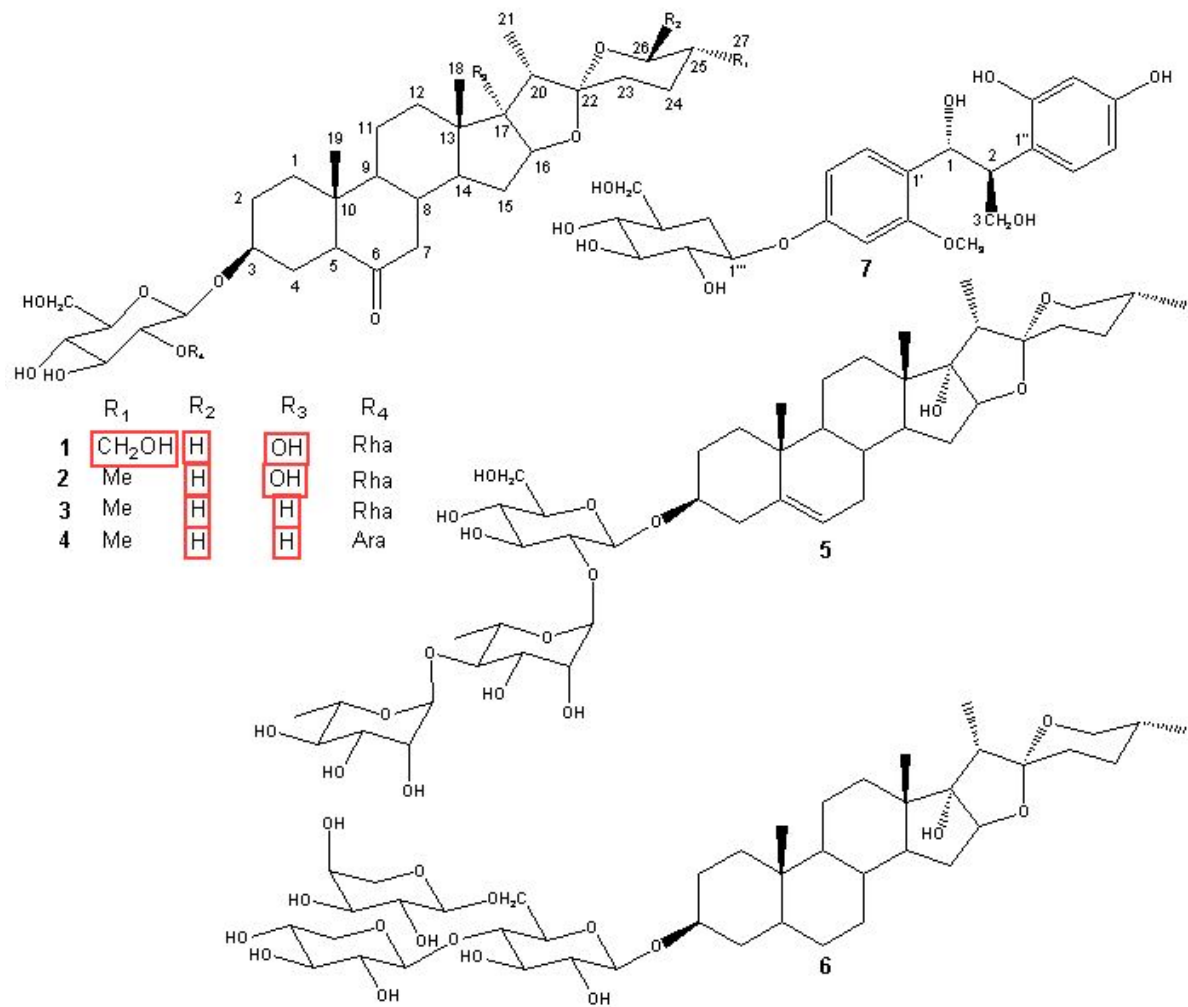

**Figure S2. ROESY correlations of 1.**

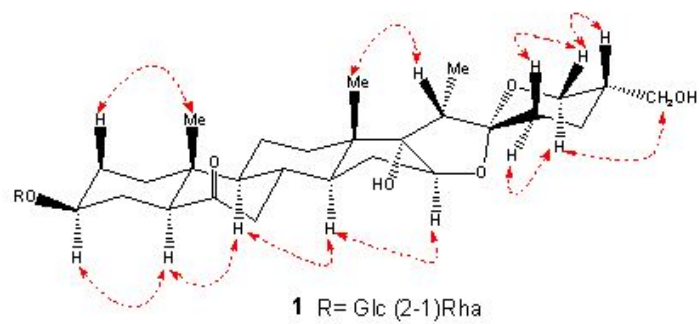

**Figure S3.** COSY and HMBC of **1** and **7**.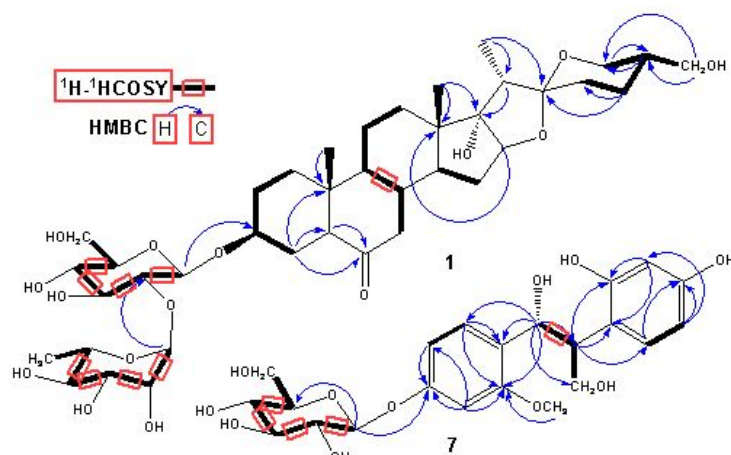

Supplement: Supplementary file 1 [file molecules-17-10494-s001.pdf]
